# Supplementary figures and images for: Noncanonical Wnt/Ror2 Signaling Regulates Basal Cell Fidelity and Branching Morphogenesis in the Mammary Gland
Source: bioRxiv. 2025 Feb 25:2025.02.25.640099. Preprint. [Version 1] doi: 10.1101/2025.02.25.640099 (PMC11888327; doi:10.1101/2025.02.25.640099)

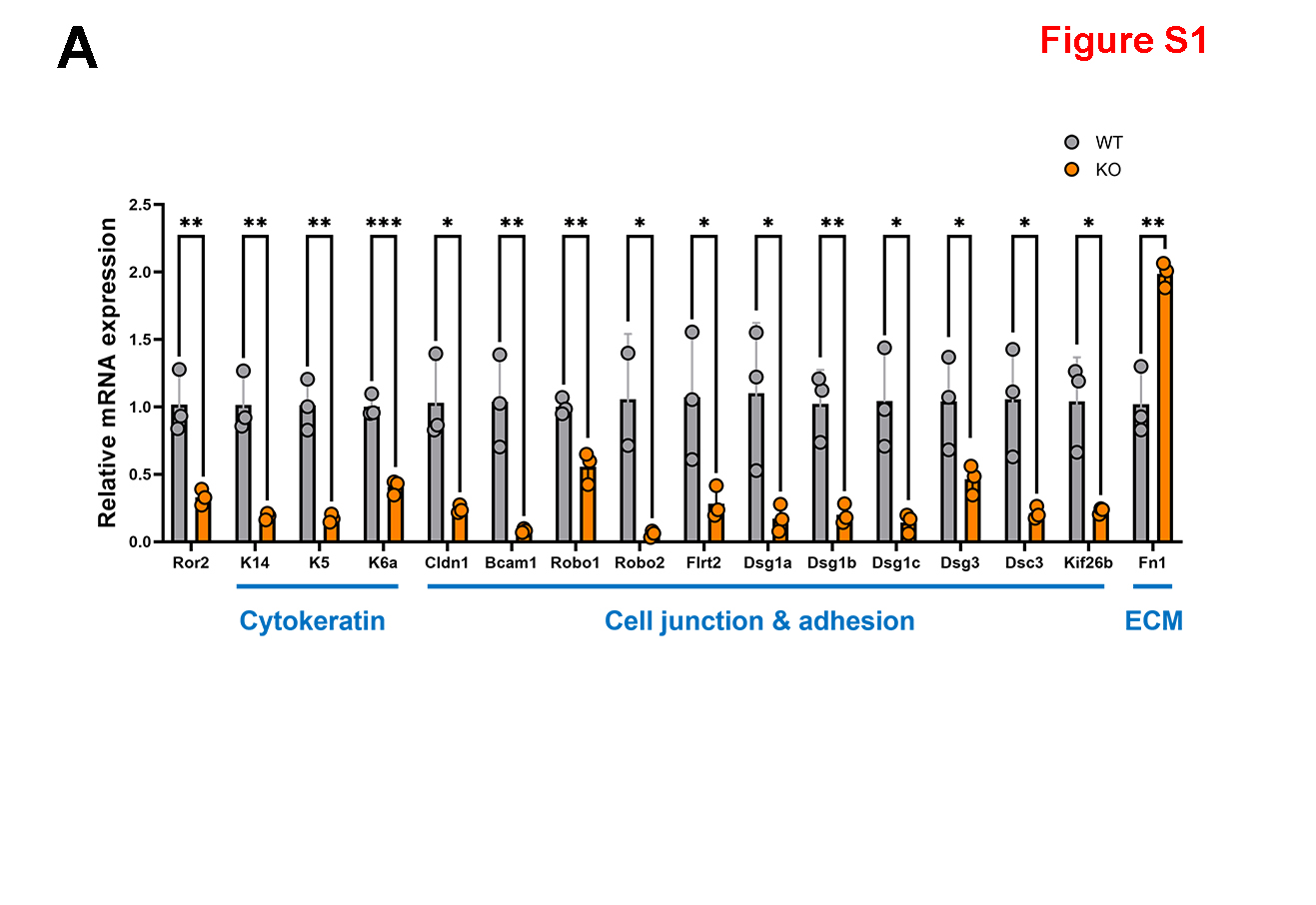

Supplement: Supplement 1 — Figure S1. Loss of Ror2 disrupts the expression of genes associated with cytokeratin, cell junctions, adhesion, and extracellular matrix (ECM) remodeling. Quantitative RT-qPCR analysis of relative mRNA expression for genes involved in cytokeratin organization (K14, K5, K6a), cell junction and adhesion (Cldn1, Bcam1, Robo1, Robo2, Flrt2, Dsg1a, Dsg1b, Dsg1c, Dsg3, Dsc3, Kif26b), and ECM regulation (Fn1) in mammary epithelial cells from p63-Ror2-WT (gray) and p63-Ror2-KO (orange) mice. Gene expression levels were normalized to GAPDH, and data are presented as fold change relative to the control group. Statistical significance is denoted by *p<0.05, **p<0.01, and ***p<0.001. n=3 biological replicates per group. [file media-1.jpg]

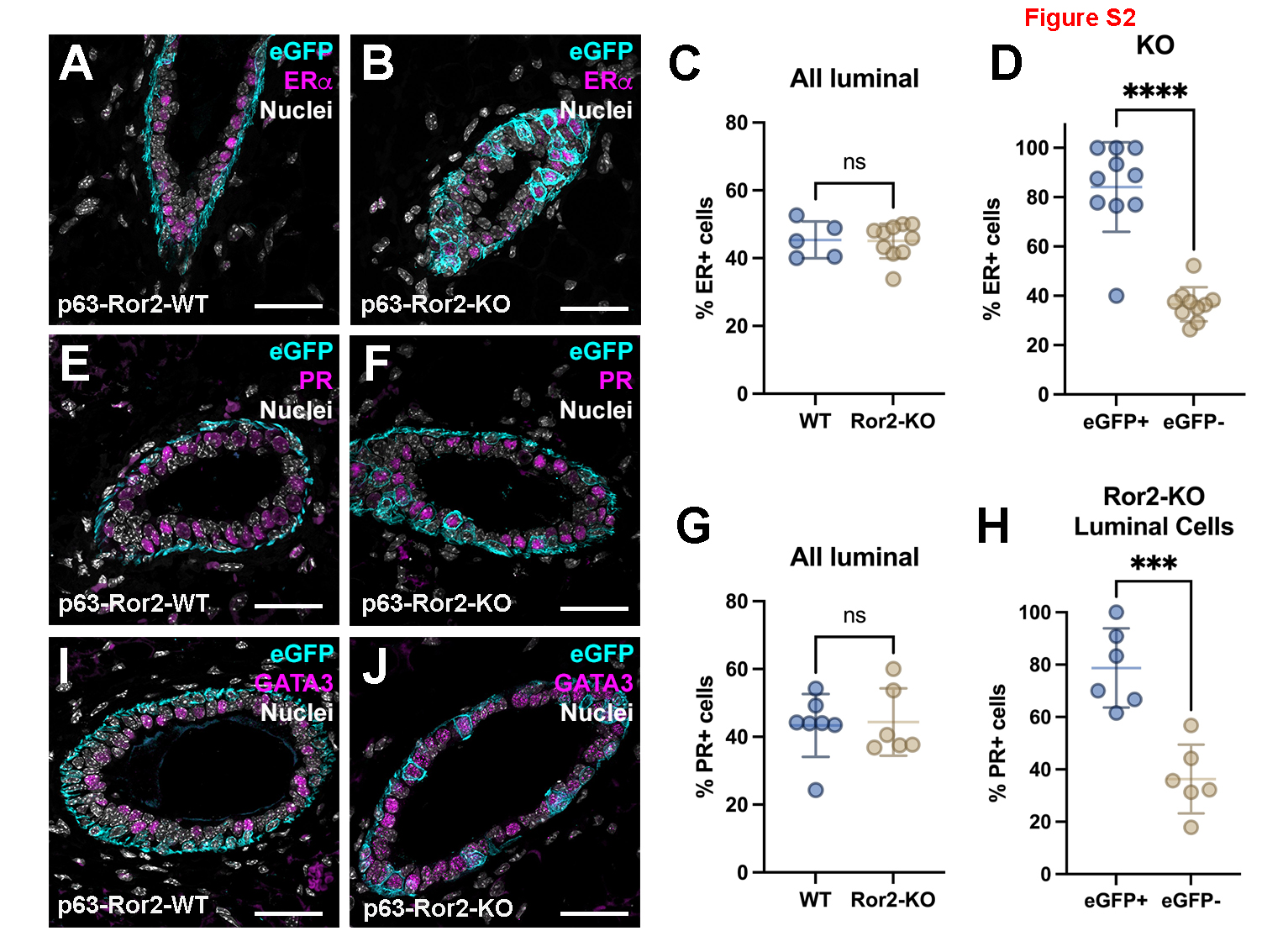

Supplement: Supplement 2 — Figure S2. Loss of Ror2 in basal cells drives a shift towards luminal cell identity. (A-B) Representative confocal images of mammary ducts from (A) p63-Ror2-WT (B) p63-Ror2-KO mice at 6 weeks old immunostained for estrogen receptor alpha (ERα, magenta), eGFP (cyan), and nuclei (gray). Scale bar, 50 μm. (C-D) Quantification of the percentage of ERα+ cells among all luminal cells in (C) WT and Ror2-KO mice (p = 0.92; n = 5 and 10 random regions from three mammary glands, respectively) and (D) among eGFP+ and eGFP− luminal cells in Ror2-KO mice (p < 0.0001; n = 10 and 10 random regions from three mammary glands). Statistical significance: ns = not significant, ****p<0.0001. (E-F) Representative confocal images of mammary ducts from (E) p63-Ror2-WT and (F) p63-Ror2-KO mice immunostained for progesterone receptor (PR, magenta), eGFP (cyan), and nuclei (gray). Scale bars, 50 μm. 6 weeks of age (G-H) Quantification of the percentage of PR+ cells among all luminal cells in WT and Ror2-KO mice (p = 0.85; n = 7 and 6 random regions from three mammary glands, respectively). (G) and among eGFP+ and eGFP− luminal cells in Ror2-KO mice (p = 0.0004; n = 6 and 6 random regions from three mammary glands). (H). Statistical significance: ns = not significant, ***p<0.001. 6 weeks of age (I-J) Representative confocal images of mammary ducts from p63-Ror2-WT (I) and p63-Ror2-KO (J) mice immunostained for GATA3 (magenta), eGFP (cyan), and nuclei (gray). Scale bars, 20 μm. 6 weeks of age. [file media-2.jpg]
